# Motion Capture Quantification of User Variation in Topical Microparticle Application

**DOI:** 10.3389/fphar.2020.01343

**Published:** 2020-09-08

**Authors:** Aaron J. Snoswell, Miko Yamada, Giles T. S. Kirby, Surya P. N. Singh, Tarl W. Prow

**Affiliations:** ^1^ Dertmatology Research Centre, The University of Queensland Diamantina Institute, The University of Queensland, Brisbane, QLD, Australia; ^2^ Robotics Design Lab, School of Information Technology and Electrical Engineering, The University of Queensland, Brisbane, QLD, Australia; ^3^ Future Industries Institute, University of South Australia, Mawson Lakes, SA, Australia; ^4^ Intuitive Surgical, Sunnyvale, CA, United States

**Keywords:** motion capture, transdermal delivery, microparticles, skin disease, volunteer study

## Abstract

Motion capture has the potential to shed light on topical drug delivery application. This approach holds promise both as a training tool, and for the development of skin technology, but first, this approach requires validation. Elongated microparticles (EMP) are a physical delivery enhancement technology that relies on a user working in the microparticles using a textured applicator. We used this approach to test the hypothesis that motion capture data can be used to characterize the topical application process. Motion capture was used to record participants while applying a mixture of EMP and sodium fluorescein to ex-vivo porcine skin samples. Treated skin was assessed using reflectance confocal and fluorescence microscopy. Image analysis was used to quantify the microparticle density and the presence of a fluorescent drug surrogate, sodium fluorescein. A strong correlation was present between applicator motion and microparticle and drug delivery profiles. There were quantitative and qualitative differences in the intra- and inter- user application methods that went beyond the level of training. Frequency and velocity of the applicator motion were key factors that correlated with EMP density. Our quantitative analysis of an experimental dermatological device supports the hypothesis that self-application may benefit from some form of digital monitoring or training with feedback. Our conclusion is that the integration of motion capture into experimental dermatological research offers an improved and quantifiable perspective that could be broadly useful with respect to topical applications, and with respect to the instruction provided to patients and clinicians.

## Introduction

There have been several studies demonstrating variabilities in topical applications (Singh and Morris, 2011; Wiedersberg and Guy, 2014). For instance, the accurate self-dosing of a given formulation can be difficult for individual patients to control. Variabilities in quantities or coverage area caused by application can lead to variabilities in efficacy and toxicity. For example, Solasso et al. showed inter- and intra- individual variabilities in pharmacokinetics of a transdermal fentanyl patch in 28 cancer patients with chronic pain (Solassol et al., 2005). As early as 1964, it was shown by Schlagel and Sanborn that instruction plays a major role in achieving repeatable and consistent application (Schlagel and Sanborn, 1964), however this requires knowledge of optimal delivery methods, which may be lacking in research literature. For example, sunscreen is one of the most common regulated topical products on the consumer market, however controversy still exists about optimal application and delivery methods (Yamada et al., 2020). Sunscreen labels often specify the quantity (1 mg/cm^2^) and interval of application (every 2 h outdoors) based on USA Food and Drug Administration sunscreen guidelines, but many people apply too little sunscreen or forget to re-apply (Petersen and Wulf, 2014). Furthermore, patient application compliance for topical drugs varies with individual demographic factors such as age, and topical absorption varies with individual biological factors (Levin and Maibach, 2012). There is a gap in our capacity to quantify application parameters, beyond measuring the amount of a formulation present after application.

Transdermal delivery is a common and effective approach used in a variety of medical and cosmeceutical fields (Yamada and Prow, 2020). One of the key challenges is getting the active compound through the skin barrier (Munch et al., 2017). Topical treatments are generally combined with chemical and physical enhancers to deliver the actives through the skin. Examples of chemical enhancers are alcohol, dimethylsulphoxide, ethylenediaminetetraacetic acid and salts (Newton, 2013). Our previous publication summarized the current physical enhancement technology available in consumer facing markets (Yamada and Prow, 2020). There are only a handful of drug delivery devices that have been commercially successful. One of the key features of a successful topical drug delivery technology is consistent drug concentration at the target tissue. The formulation, drug properties and application are all critical factors contributing to the consistency of pharmacokinetics and pharmacodynamics.

In the present work, we use a physical enhancement technology, elongate microparticles (EMP), as a case study to evaluate motion capture as a means to generate useful information on the topical application process. EMPs are unbound high aspect ratio microparticles that penetrate the skin at low angles from the surface. In our protocol, EMPs are applied topically using a custom 3D printed “pen-like” applicator with a single-use, disposable, micro-textured head (Raphael et al., 2014; Raphael et al., 2015). This approach is minimally invasive and does not damage the skin, which differentiates it from dermabrasion approaches that use sea sponge spicules. We have previously used EMP technology to enhance the delivery of a range of active ingredients in excised and volunteer skin (Raphael et al., 2014; Yamada et al., 2018). We hypothesized that the kinematic motion of a topical application may help characterize the effectiveness of drug delivery. As such, the aim of this study was investigate and characterize quantitative correlations between application motion and drug and/or EMP uptake.

Assessing topical drug delivery depth and distribution can be determined by non-invasive imaging modalities including fluorescent light microscopy, confocal microscopy, optical coherent tomography, and reflectance microscopy if the active ingredient has the prerequisite properties (Gregoire et al., 2019; Pena et al., 2020). To investigate our hypothesis, we applied a fluorescent drug phantom with the EMP delivery enhancer, then used RCM and fluorescence imaging to determine the EMP density and drug distribution profile across the treatment area. We recorded kinematic motion capture data (N=20) using a micro-particle applicator shaped like a pen. These data were then analyzed based on the corresponding EMP density at the target site. Summary statistics and Ordinary Least Squares regression models were used to analyze the motion and imaging data after application. Some application parameters were found to strongly correlate with EMP and drug deposition profiles. We found that EMP density per mm^2^ could provide numerical data for transdermal delivery. There were some correlations between number of EMP and intensity of NaF. Statistical analysis revealed that applicator velocity was closely correlated with delivery parameters, suggesting this motion feature is key for characterizing topical delivery. Other parameters, like azimuth and movement frequencies, appeared to have less correlation with EMP density and drug distribution. Our conclusion is that motion capture has the immediate potential to quantify user application parameters that may inform formulation design, device development and the generation of training instructions. In the future, we see this approach maturing into a tool that give active real-time application feedback and quantitative application data for academic, industry and ultimately clinical use that will decrease efficacy variation and improve patient care.

## Materials and Methods

### Reagents

Sodium fluorescein (Retinofluor, #INJ140, Phebra, Australia).

### Elongate Microparticle Fabrication

The EMPs were fabricated in-house using micro-chopping. A layer approximately 1 mm deep of 8 µm dimeter silica oxide filament was processed to obtain 173.3 ± 100.8 µm EMP, as described in our previous work (Raphael et al., 2014). We then measured the particle length distribution using visible light microscopy, followed by image analysis.

### Elongated Microparticle Applicator Fabrication

**Figure 1:**
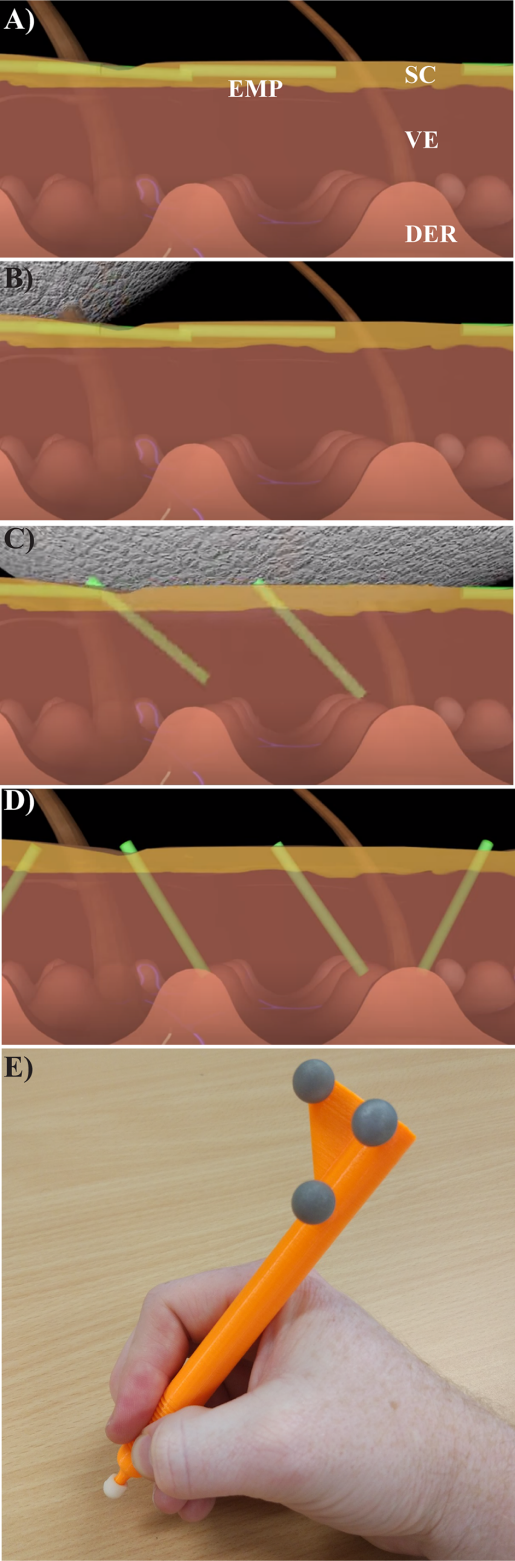
*Using the textured applicator.* **(A)** Unbound elongate microparticles (green, EMPs) are shown after being placed on the skin surface along with a topical drug compound. **(B, C)** A specially designed textured application device is used to gently massage the EMP solution into the skin, causing EMP penetration into the *stratum corneum* (SC) and viable epidermis (VE), but not the dermis (DER). **(D)** The EMPs allow for increased drug uptake, and naturally leave the skin through trans-epidermal elimination over the course of several days to three weeks (Raphael et al., 2014). **(E)** The instrumented, rigid EMP application device is shown with optical motion capture markers (gray spheres).

Autodesk Inventor Professional 2014 was used to design the custom “pen” type applicator with attachment points for optical motion capture instrumentation (**Figure 1E**), and to design the applicator heads. The file was exported in the stereolithography (STL) format and 3D-printed using a MakerBot Replicator 2 desktop 3D printer with Polylactic (PLA) plastic material. The single-use mechanical textured applicator heads were 3D-printed using a V-Flash FTI-GN Material. Post processing of the applicator heads was done using the corresponding V-Flash protocol.

### Textured Applicator Use

The textured applicator device was developed as an effective method of delivering EMPs through the viable epidermis without damaging the skin surface (Raphael et al., 2014). Without application, the EMPs lie flat against the surface of the skin as depicted in **Figure 1A**. The applicator is used (as shown in the sequence of **Figures 1B–D**) to push the EMPs into the skin, thus enhancing drug penetration. This applicator was developed using rapid prototyping techniques and features motion capture reflectors (**Figure 1E**). Reflectance Confocal Microscopy (RCM) was used to gather additional data regarding the EMP distribution per mm^2^.

### Topical Application by Volunteers

A convenience sample of five volunteers participated in the experiment. The volunteers had varying levels of expertise applying EMPs with our mechanical application protocol, ranging from substantial experience to no experience. Each volunteer performed five replicate applications.

Frozen porcine skin samples were thawed, shaved and washed in preparation for use in this experiment. Methylene blue stain was used to identify and avoid damaged skin, and 2 cm diameter circular target sites were marked on the skin surface. Before each application, an unused textured applicator head was attached to the application device and the motion capture system was re-calibrated. Pre-weighed EMP (25 mg) and 200 μl of NaF solution (250 μg/mL) were applied to the center of each site (poured directly from a test-tube, then delivered using a pipette respectively). Each participant then immediately massaged the combined solution using the mechanical applicator device for 30 seconds. They then repeated the other replicates at different locations on the skin sample.

The rigid application device was instrumented to enable optical kinematic motion capture. An OptiTrack V120 Trio commercial motion capture system (NaturalPoint Inc., USA) was used to record the kinematic motion trajectories of the rigid body applicator at approximately 120 Hz. Porcine skin was photographed before application using a visible-light digital SLR camera, and post-application using a Typhoon fluorescing scanner (Typhoon FLA9500, GE Healthcare Life Sciences) to record NaF intensity. Reflectance Confocal Microscope (RCM) (excited at 750 nm) (Vivascope 1500 Multilaser, Lucid Inc., USA) was used to record 2000 × 2000 × 50 μm stacks at 5 μm depth steps for five equally spaced samples from each application site (centrally located, as well as in each of the relative cardinal directions).

### Motion Capture and Motion Trajectory Analysis

Application parameters were assessed by 3D motion capture using an OptiTrack V120 Trio. The data were collected using a 3D printed applicator with calibrated, reflective markers. The users applied EMPs to excised skin while 3D tracking information was being collected at 120 frames per second. The 3D application motion parameters were then extracted. The recorded motion trajectories were inspected, trimmed and manually cleaned using the OptiTrack Motive software (NaturalPoint Inc., USA). Of the original 25 recordings, two were discarded due to the application being interrupted, and three were discarded due to significant line-of-sight occlusion corrupting the motion capture data. Valid recordings were exported as N=20 motion trajectories ranging from 8.6 to 30.3 seconds in duration. The Computer Aided Design (CAD) model of the applicator device was used to transform the raw motion capture data to a right-handed skin-centric coordinate system with the +X (rightward) and +Z (upward) axes lying in the plane of the skin surface aligned relative to the center of each application site. The coordinate system used for analysis is summarized in **Figure 3C**. The motion trajectory data were analyzed using Python 3.6.5 and Stata SE 16.0 (StatCorp Inc., USA).

### Fluorescence and Reflectance Imaging and Analysis

The treated skin was then assessed for microparticle number per mm^2^ using Reflectance Confocal Microscopy. RCM data were generated using 785 nm excitation laser at 0–5 Mw as previously published (Raphael et al., 2014). Individual z-stacks consisted of horizontal 500 × 500 µm optical sections acquired at every second micron from the skin surface to a depth of 200 µm. Reflectance images were processed in ImageJ (Java based freeware, National Institutes of Health, USA) in 8-bit gray scale mode. The z-stack images were used to identify EMP distribution. Fluorescence scanning at 488 nm enabled a visual overview of fluorescence (NaF) density on the skin surface (Typhoon, GE Healthcare). From fluorescent images, the mean intensity of each treated site was calculated. Summary statistics from the image data were calculated using GraphPad Prism 5.03 software (GraphPad Software Inc., USA).

## Results

### EMP Density and NaF Fluorescence Intensity

**Figure 2:**
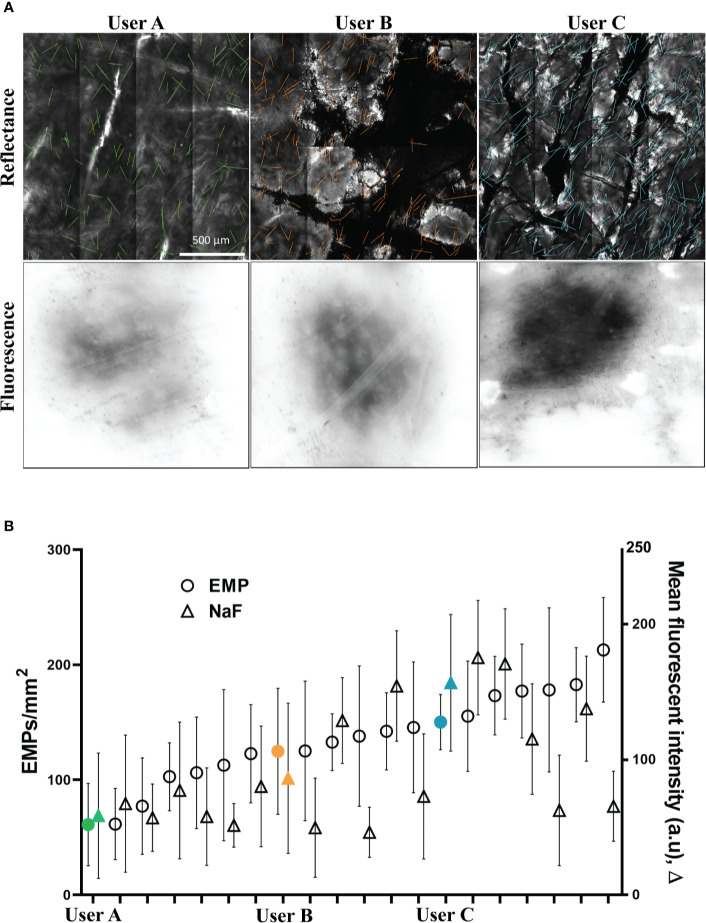
**(A)** Representative images of porcine skin imaged using RCM (Reflectance) and conventional inverted fluorescence microscopy (Fluorescence). **(B)** EMP and NaF data from 20 replicates shown as mean and standard deviation. Users A (low EMP/mm^2^), B (moderate EMP/mm^2^) and C (high EMP/mm^2^) are solid and marked in Green, Orange and Blue, respectively, and this color coding remains consistent throughout.

Two key parameters were quantified to determine the effectiveness of topical delivery. These were the number of EMP per mm^2^ (determined from RCM) and the intensity of surface NaF determined by integrating the fluorescence intensity over the application site area. Representative images from three users are shown in **Figure 2A**. Quantified values for the three users are shown in **Figure 2B** along with a series of other users that were similar to our prior publications using human skin (Raphael et al., 2014). These users were sorted by EMP in ascending order to present them as a spectrum of less effective (left) to most effective (right) delivery. Discrepancies between EMP density and NaF intensity are clearly evident for some users. Users A, B and C represent a cross section of low, medium, and high EMP per mm^2^ respectively, and results from these users were selected for further technique analysis. The user colors remain consistent throughout; green-low (SD = ± 35.7), orange-moderate (SD = ± 54.7) and blue-high (SD = ± 24).

### 3D Motion Capture Reveals Discrete Differences Between Users

We observed that the captured motion data contained both high-frequency and time-varying characteristics. As such, we analyzed the motion data using both time- and frequency-domain features. We identified time- and frequency-domain properties of the motion to serve as predictor features in Ordinary Least Squares regression models. These regression models were developed using both EMP density and NaF intensity as outcomes to assess the predictive power of the kinematic motion characteristics. The regression models are summarized in **Table 1**.

**Table 1:** Relationships between motion capture outputs and NaF intensity.

NaF outcome	Motion capture model type	Adjusted R^2^	Motion capture feature	Correlation coefficient (R^2^) (95% CI)
NaF Intensity Mean (Fraction 0 – 1)	Time-domain	0.76^**^	Velocity (Inter-Quartile Range)	0.70^**^ (0.51 to 0.88)
	Frequency-domain	0.79^**^	1 Hz Power	−0.58^*^ (−1.03 to −0.13)
			4 Hz Power	0.43^**^ (0.15 to 0.70)
			11 Hz Power	2.39^**^ (1.71 to 3.05)
NaF Intensity Standard Deviation	Time-domain	0.43^**^	Velocity (median)	−0.35^**^ (−0.56 to −0.13)
			Azimuth (median)	−0.20^**^ (−0.30 to −0.09)

*indicates 95% confidence level; **indicates 99% confidence level.

**Figure 3:**
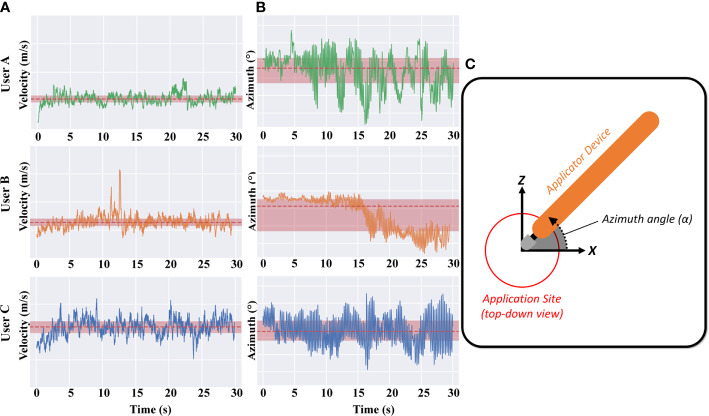
**(A)** Applicator velocity magnitude is shown for the example “high”, “medium”, and “low” applications (Users A, B and C respectively). Note that the y-axis limits and ranges for all plots are consistent. **(B)** Applicator azimuth angle for the example “high”, “medium”, and “low” applications (Users A, B and C respectively). **(C)** Inset shows the azimuth angle – azimuth values further from zero correspond to users twisting their wrist/hand to rotate the applicator away from/closer to their torso.

**Figure 3A** shows the velocity of the applicator for users A, B and C, with the mean and +/− one standard deviation indicated in red. We found that the tip velocity had a strong correlation with EMP and NaF delivery (R^2^ 0.79).

The azimuth of the applicator for users A, B and C is also shown in **Figure 3B**, with the mean and +/− one standard deviation indicated in red. The azimuth angle is essentially the angle of the tip with respect to the surface of the skin – azimuth values further from zero correspond to users rotating their wrist in the plane of the bench-top, towards/away from their torso. Whilst the azimuth of the applicator is clearly different between users, no discernible correlation is observed with delivery effectiveness. Differences can be seen between the three users but there were only weak correlations between this and the density of EMP in skin. Users A and C exhibit a slight oscillation every couple of seconds whereas user B remains fairly consistent with a drop of tip angle at 15 s (**Figure 3B**, middle panel). Since the EMP applicator tip was developed to work effectively at different angles, it is reassuring and unsurprising that applicator azimuth had little effect.

We also analyzed the motion using frequency-domain features. The average spectral power magnitude in 1 Hz bins at frequencies from 1 Hz to 13 Hz were selected predictor variables for regression analysis. Above 13Hz, the spectral power was approximately flat (data not shown). Higher-frequency spectral power indicates rapid cyclic motion of the applicator to and from the center of the application site. The normalized spectral power magnitude (mean and ± one standard deviation) for high and low EMP density applications was analyzed to identify relationships between motion and NaF intensity (**Table 1**).

## Discussion

For over 35 years, researchers have been developing novel transdermal approaches with varying level of success by focusing on chemical properties of active ingredients and skin properties (Wiedersberg and Guy, 2014). *Stratum corneum*, viable epidermis and dermis properties can impact significantly on percutaneous absorption (Newton, 2013; Munch et al., 2017; Szunerits and Boukherroub, 2018), however very little work has investigated the individual physical application process. This is likely due to widely established analytical techniques available to study small molecules and skin properties, in contrast to the relative absence of technologies to study human movement during the application of topical drugs (Singh and Morris, 2011).

This is a knowledge gap in a topical drug field, especially considering the lack of clarity regarding inter-individual variation in transdermal application. For example, Levin and Maibach compared the inter-individual variation in transdermal and oral delivery, finding that transdermal drug delivery has more favorable pharmacodynamic and pharmacokinetic profiles with a decreased C_max_, increased time at C_ss_, and longer life-time, depending on the active ingredient. However they highlight the need for further investigation to better characterize the nature of inter-individual variation (Levin and Maibach, 2012). Ours is the first study to utilize motion capture technology to quantify inter-individual variation in human movement during the application process of an experimental delivery enhancement technology.

Another study showed the cure rates for non-melanoma skin cancer ranged from 65% to 100% for topical imiquimod and 61% to 92% for 5-flourouracil (Chitwood et al., 2013). It is unclear what role the application process plays in this high level of variability. A clinical trial investigating topical 5-fluorouracil efficacy in the treatment of actinic keratosis revealed similar levels of variation. Three different groups conducted the same protocol where patients were self-medicated with 0.5% topical 5-fluorouracil twice a day for three months. The clinical clearance rates from these three studies were 74.5%, 62% and 55.2% respectively (Stockfleth et al., 2011; Simon et al., 2015; Nguyen and Rivers, 2016). After six months post-treatment, there was 40% clinical recurrence of actinic keratosis observed (Stockfleth et al., 2011). These are examples where individual kinematic application differences, in combination with individual skin variation, may have confounded these results. We believe that the learnings from motion capture technology may help disentangle causal factors contributing to the relatively high level of variability observed with topical drug delivery compared to other delivery routes (Levin and Maibach, 2012).

Our results show for the first time that that qualitative inter-individual variation in behavior (specifically, kinematic application motion) can be characterized and correlated with quantitative treatment outcomes. For example during data collection, we qualitatively observed volunteers adjusting their grip on the mechanical applicator, or adjusting hand pose during application (e.g. User B in **Figure 3B** at 15 s), however subsequent analyses revealed that this particular mode of variation (change in azimuth) had low correlation with drug delivery outcomes. In this case, these data confirmed that our topical applicator device design in fact met a key design goal of correct functioning regardless of azimuthal variation. The motion capture data also served to spur discussion with users regarding such changes in their behavior mid-application, revealing that ergonomic properties of the applicator device contributed to the likelihood of mid-application adjustments. Thus, motion capture may serve as a useful tool in the design of topical delivery devices.

We further envisage that motion-capture technology may also be useful to inform treatment protocols. We first described the application of EMP in porcine flank skin in 2013 (Raphael et al., 2013), there observing an EMP density of 41 ± 11 EMP per mm^2^. This is similar to User A in our study, i.e. 61 ± 35 EMP per mm^2^. Both studies utilized similar aged animals and the same body site, but the Raphael et al. study utilized a single individual applying the EMP, whereas here we sought to characterize the application behavior of multiple users. It is possible that we could have significantly increased the delivery enhancement in our previous work had we utilized motion capture technology to refine the application technique prior to that study.

Our results here were using an ex-vivo porcine model, however in 2014, we described the use of EMP in human skin showing similar results to User A in this study, *viz.* 76 ± 40 EMP per mm^2^ (Raphael et al., 2014). This suggests our motion capture analysis and results may be applicable in human skin, however further experimentation is necessary to validate this point. We also note that usage of motion capture in a clinical setting may present procedural and technological challenges. *E.g.* we had to discard some data due to our volunteers moving their body between the topical applicator device and the motion capture system, obscuring the line-of-sight which is necessary for tracking with optical motion capture. This complicating factor could perhaps be addressed in future work by utilizing different motion capture technologies. Furthermore, our study was performed with skin samples pinned to a laboratory bench-top, which is not possible in a clinical setting. As such, we recommend that motion capture technology should be carefully integrated, taking into account the topical application environment, site, user, and the application protocol.

This feasibility study demonstrates the use of motion capture for the analysis of topical application parameters between individuals. This approach has value by improving our understanding of the mechanical differences between individuals utilizing a pen type applicator. We believe this approach also holds value for studying topical application in general, especially where there are significant variations in pharmacological outcomes or clinical efficacy. It is our opinion that motion capture of topical drug application processes is complementary to conventional pharmacokinetic and pharmacodynamic experimental designs, and holds great promise in device and treatment protocol design.

## Data Availability Statement

The raw data supporting the conclusions of this article will be made available by the authors, without undue reservation.

## Ethics Statement

All human participants were consented into this study before participation. Human and animal ethics approval for this study was granted by the University of Queensland human ethics committee, approval number HREC/12/QPAH/216.

## Author Contributions

TP sourced funding for the study and designed the study protocol. AS completed the instrumented applicator design, data collection and data analysis. SS assisted with the applicator instrumentation and data analysis. MY completed image analysis. All authors contributed to the article and approved the submitted version.

## Funding

We acknowledge NHMRC grant APP1065802 (TP), NHMRC Fellowship APP1109749 (TP). We also acknowledge the financial support from an Australian Government Research Training Program Scholarship. The 3D motion capture instrument was partially supported by Australian Research Council Discovery Project DP160100714 (SS).

## Conflict of Interest

At the time of writing, Author SS was employed by the company Intuitive Surgical.

The remaining authors declare that the research was conducted in the absence of any commercial or financial relationships that could be construed as a potential or actual conflict of interest.
